# Maxillofacial growth changes after maxillary protraction therapy in children with class III malocclusion: a dual control group retrospective study

**DOI:** 10.1186/s12903-023-03790-6

**Published:** 2024-01-03

**Authors:** Shukui Xu, Yang Liu, Yan Hou, Yinghui Li, Xiaolei Ge, Linna Wang, Liru Zhao, Wensheng Ma

**Affiliations:** 1 Department of Orthodontics, Hebei Key Laboratory of Stomatology & Hebei Clinical Research Center for Oral Diseases, School and Hospital of Stomatology, Hebei Medical University, Shijiahzuang, 050017 China; 2 Department of Orthodontics, School of Medicine, Tianjin Stomatological Hospital, Nankai University, Tianjin, 300041 China; 3 Tianjin Key Laboratory of Oral and Maxillofacial Function Reconstrution, Tianjin, 300041 China

**Keywords:** Maxillary protraction therapy, Skeletal class III malocclusion, Stability, Growth, Orthopedic treatment

## Abstract

**Purpose:**

To investigate the balance between post-treatment effect and continued nature growth after maxillary protraction treatment in patients with skeletal class III malocclusion.

**Methods:**

31 patients aged 8.79 ± 1.65 years with skeletal Class III malocclusion had been treated with maxillary protraction and the treatment lasted an average of 1.16 years. The average observation duration after treatment in the maxillary protraction group was 2.05 ± 0.39 years. In the control groups, a sample of 22 patients (9.64 ± 2.53 years) with untreated skeletal class III malocclusion and 24 patients (9.28 ± 0.96 years) with skeletal class I malocclusion were matched to the treatment group according to age, sex and observation period. The mean observation interval of the control groups was 2.39 ± 1.29 years in the class III group and 1.97 ± 0.49 years in the class I group.

**Results:**

The active orthopedic treatment effect showed a opposite trend to the natural craniomaxillofacial growth effect after treatment in many aspects. In the observation duration of treatment group, decrease in ANB, Wits appraisal and BAr-AAr were statistically significant compared to class I control group (p < 0.001), and there was a significant increase in NA-FH (P < 0.001) which was contrary to class III control group. Treatment group presented a significant increase in Gn-Co (P < 0.01) and Co-Go (P < 0.001), except for changes in the extent of the mandibular base (Pog-Go, P = 0.149) compared to class I control group. The vertical maxillomandibular skeletal variables (Gonial; MP-SN; MP-FH; Y-axis) in treatment group decreased significantly compared to those in class III control group (P < 0.01). U1-SN and L1-MP showed a significant increase, which was similar to the class I group (P > 0.05), and overjet decreased significantly relative to both of the two control groups (P < 0.05).

**Conclusion:**

Maxillary protraction therapy led to stable outcomes in approximately 77.42% of children with Class III malocclusion approximately 2 years after treatment. Unfavorable skeletal changes were mainly due to the greater protrusion of the mandible but maxillary protraction did have a certain degree of postimpact on the mandibular base. Protraction therapy does not fundamentally change the mode of maxillary growth in Class III subjects except for the advancement of the maxilla. Craniomaxillofacial region tend to restabilize after treatment and lead to skeletal growth rotation and more dentoalveolar compensation.

## Background

Skeletal class III malocclusion is a common pathological phenomenon in clinical orthodontics and is characterized by maxillary retrognathism or mandibular prognathism alone, or these two situations can exist simultaneously [1]. Recently, some studies have achieved a consensus on early interventions for the development of skeletal class III malocclusion, and a series of orthopedic treatment approaches have been proposed [2, 3]. However, clinicians do not always have the chance to modify a patient’s aberrant Class III growth pattern at an early age, and relapse following orthopedic treatment continues to be a major clinical issue, as the long-term stability of maxillofacial growth improvement for skeletal class III malocclusion patients has inherent uncertainty [4, 5]. Given this, growth represents the main component of the uncertainty in class III malocclusion subjects, whether it is undergrowth of the maxilla or overgrowth of the mandible, as seen in those who relapsed severely, eventually leading to the final treatment option of orthognathic surgery in adults [6].

Maxillary protraction is one of the commonly used clinical approaches for skeletal class III malocclusion in young patients as a kind of dentofacial orthopedic treatment [7, 8], causing anterior movement of the maxilla and restricting or redirecting mandibular growth to some extent, leading to a clockwise rotation trend of the mandible and reducing the compensatory effects of dental inclinations [9, 10]. However, the remaining skeletal remodeling effect after maxillary protraction remains controversial. Several studies have indicated that the early use of maxillary protraction may have favorable stability, with a stable rate of maxillary bone growth after lifting the restriction from negative anterior overjet [11, 12]. While some studies have questioned whether the development of skeletal class III malocclusion can be changed and the precise growth mechanisms after maxillary protraction, they have indicated that excessive growth of the mandible after removal of the restrictive counterforce from maxillary protraction may cause relapse of the sagittal jaw-face relationship [4, 13, 14]. Therefore, it appears that the posttreatment stability of Class III malocclusion should focus on the correction of maxillary deficiency and the growth potential of the mandible.

Considering that previous studies often focused only on the possibility of relapse after treatment by comparing with skeletal class III control group. Notably, a continued nature growth will still exists which is attributed to the fact that the treatment have been basically completed before the end of growth spurt. Therefore, growth after maxillary protraction should be consider as a balance between post-treatment effect and continued nature growth, and the latter should be explored by setting skeletal class I control group. Therefore, a dual control group was set up to investigate the growth mechanisms after the removal of maxillary protraction under a more comprehensive analyse. The hypothesis of the present study was that the growth pattern after maxillary protraction would intermediate between the two control groups.

## Methods

### Study design and sample

In this retrospective study, posttreatment subjects were obtained from the Department of Orthodontics, Hospital of Stomatology, Hebei Medical University, China. The inclusion criteria at the beginning of treatment (T0) included the following: (1) ANB angle ≤ 0° and Wits appraisal ≤ − 2.0 mm; (2) Class III molar relationship; (3) anterior crossbite or edge-to-edge incisal relationship; (4) cervical stage (CVS) CVS2-CVS3; and (5) 22° ≤ MP-FH angle ≤ 32°. The exclusion criteria included the following: (1) pseudo-Class III malocclusion which is caused by premature contact with functional mandible forward positioning; (2) congenital absence, extracted, or supernumerary teeth; (3) craniofacial anomalies such as cleidocranial dysplasia or cleft lip and palate; (4) temporomandibular joint dysfunction; and (5) previous treatment. The inclusion criteria for the untreated skeletal class III control group were consistent with the selection criteria mentioned above. The inclusion criteria of the skeletal class I control group included the following: (1) ANB angle between 0° and 3°; (2) Wits appraisal between − 2.0 mm and 2.0 mm; (3) 22° ≤ MP-FH angle ≤ 32°; and (4) nonextractive orthodontic treatment with fixed appliances for moderate dental problems, including minor crowding and malpositioning of the teeth.

After screening according to the abovementioned criteria, 77 patients were finally included in the study (Table 1). Among them, 31 patients (16 males and 15 females) with skeletal Class III malocclusion (Treatment group, TG) had been treated with a combination of maxillary protraction and rapid maxillary expansion (RME). In the control group, a sample of 22 patients (12 males and 10 females) with untreated skeletal Class III malocclusion (Control group III, CG III) and 24 patients (10 males and 14 females) with skeletal Class I malocclusion (Control group I, CG I) were matched to the treatment group according to age, sex and observation period.

**Table 1:** Comparison of sex distribution in each group

		Sex		X^2^	P value
		Male	Female		
Type	Skeletal Class III malocclusion treatment group (TG) (n = 31)	16 (51.61%)	15 (48.39%)	0.866	0.648
	Skeletal Class III malocclusion control group (CG III) (n = 22)	12 (54.55%)	10 (45.45%)		
	Skeletal Class I malocclusion control group (CG I) (n = 24)	10 (41.67%)	14 (58.33%)		

*P < 0.05; **P < 0.01; ***P < 0.001

### Skeletal class III malocclusion treatment protocol and observation duration

Subjects with Class III malocclusion were treated with RME and maxillary protraction who aged 8.79 ± 1.65 years before treatment and cephalograms were taken (T0). Treatment was started by cementing a Hass expander with anterior protraction hooks at the mesial surfaces of the canines. Patients were instructed to activate the screw once (0.25 mm) or twice (0.5 mm) a day until an overexpansion of 3 mm for the dental arch width was achieved. At the same time, the protraction facemask was adjusted and placed, and patients were instructed to wear the mask more than 14 h a day. The elastics connecting the protraction hooks to the mask hooks were oriented in a downward and forward direction at an angle of approximately 30° relative to the occlusal plane. The elastics were used with a traction vector force of 300–500 g per side and were changed once a day. This treatment lasted 1.16 ± 0.52 years when a positive overjet of greater than 3 mm and a class I or class II molar relationship were achieved, after which cephalograms were again taken (aged 9.95 ± 1.67 years, T1). Subjects were revisited periodically after removal of the maxillary protraction facemask (T2). The average observation duration in the experimental group was 2.05 ± 0.39 years.

### Skeletal class III malocclusion control group observation duration

The skeletal Class III control group included patients aged 9.64 ± 2.53 years who chose to receive orthognathic surgery in adulthood and signed informed consent forms. None of the subjects had undergone prior treatment or procedures and were revisited periodically. Cephalograms were taken before observation (T1) and during the observation duration (T2). The mean T1-T2 interval was 2.39 ± 1.29 years.

### Skeletal class I malocclusion control group observation duration

To observe the development of skeletal Class I malocclusion, the treatment and retention periods for moderate dental problems were considered as the duration for observing changes in jaw relation. Orthodontic treatment consisted of the use of upper fixed appliances or the segmental arch technique to avoid settling elastics in the sagittal direction during treatment. The retention protocol involved thermoplastic orthodontic retainers made of transparent resin. Cephalograms were taken before treatment (aged 9.28 ± 0.96 years, T1) and during the retention periods (T2), and the mean interval for T1-T2 was 1.97 ± 0.49 years.

### Measurement method

Lateral cephalograms were scanned using the KaVo Dental Systems at the correct calibration and same magnification (Zoom:43.19%, WL:127, WW:255). Digital images were traced using Dolphin Imaging Software (Version 11.8), all of the lateral cephalograms measurements were done by trained personnel. Each radiograph was measured at least three times and then the average was taken to reduce the error. Method error (ME) analysis was calculated as follows: $\text{M}\text{E}=\sqrt{\frac{\sum {\text{d}}^{2}}{2\text{n}}}$ (d represents the difference between each measurement; n represents the number of double registrations). 10 measurement items were selected randomly from the cephalometric readings, ME^1^ (between the first and second measurements) and ME^2^ (between the second and third measurements) were calculated and compared by t tests. There was no significant difference between the two ME group.

**Fig. 1:**
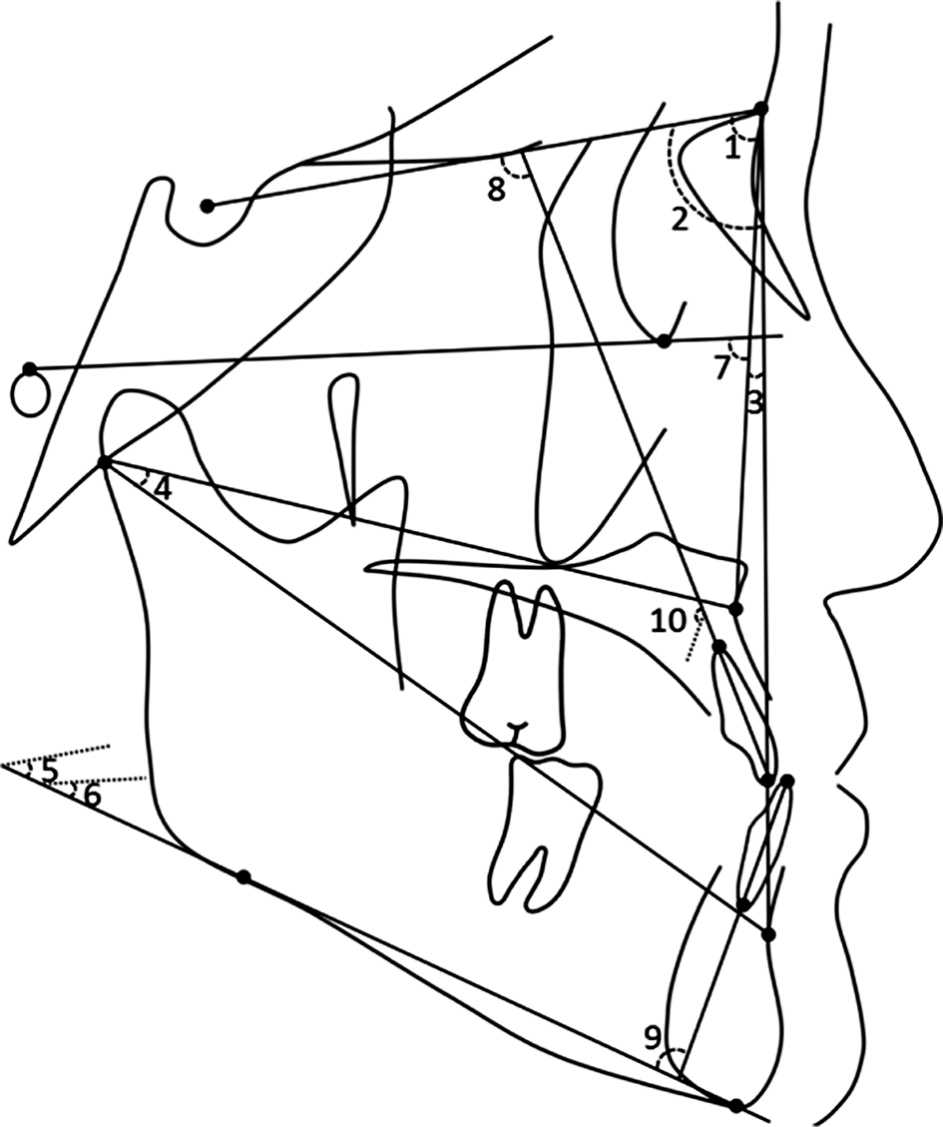
Angular measurements used in this study (1) 1. Maxillary position related to anterior cranial base (SNA angle); 2. Mandibular position (SNB angle); 3. Intermaxillary position related to nasion (ANB angle); 4. Intermaxillary position related to articulare (BAr-AAr angle); 5. Mandibular plane to anterior cranial base plane angle (MP-SN angle); 6. Mandibular plane to Frankfort plane angle (MP-FH angle); 7. Maxillary position related to Frankfort plane (NA-FH angle); 8. Inclination of upper incisor (U1-SN angle); 9. Inclination of lower incisor (L1-MP angle); 10. Interincisal angle (U1-L1 angle)

**Fig. 2:**
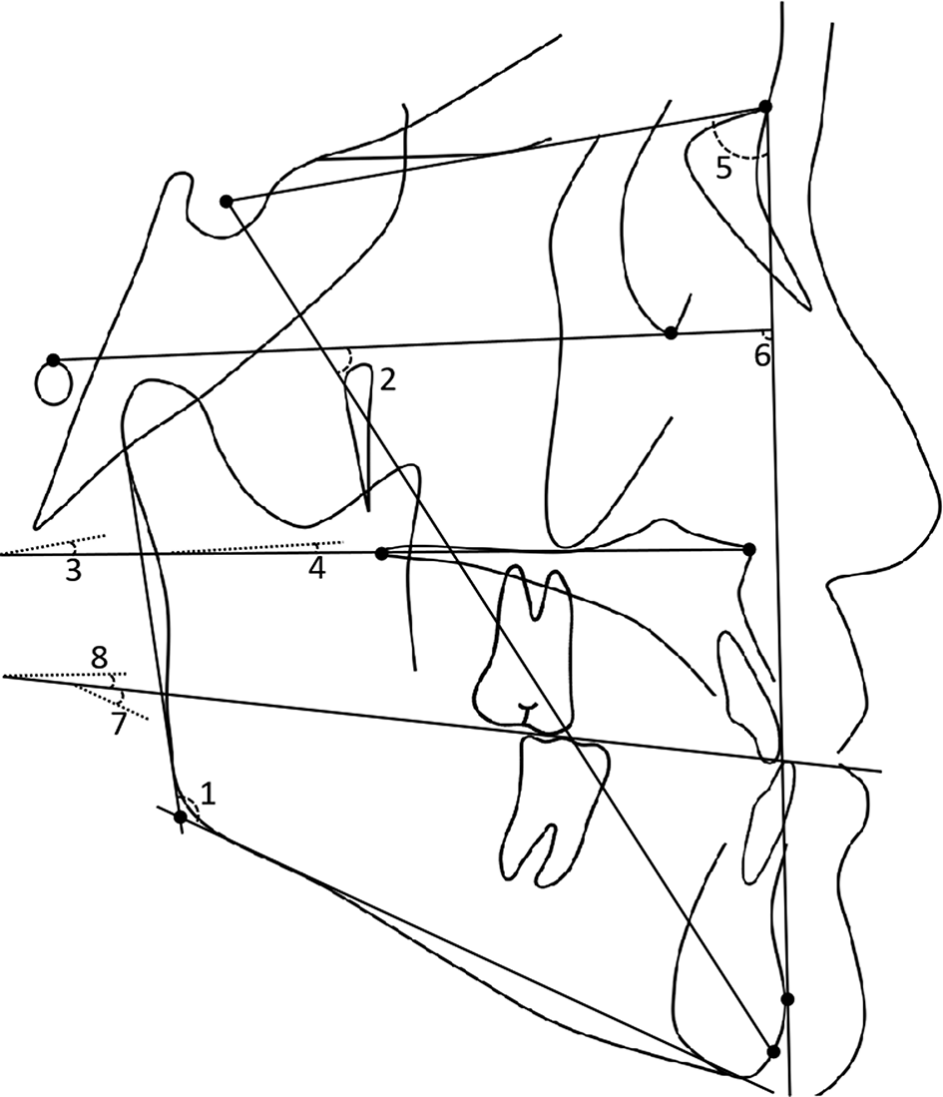
Angular measurements used in this study (2) 1. Gonial angle (Ar-Go’-Me angle); 2. Y axis angle (SGn-FH angle); 3. Angle of inclination of palatal plane related to anterior cranial base plane (PP-SN angle); 4. Angle of inclination of palatal plane related to Frankfort plane (PP-FH angle); 5. Pogonion position related to anterior cranial base (SNP angle); 6. Facial angle (NP-FH angle); 7. Occlusal plane to mandibular plane angle (OP-MP angle); 8. Occlusal plane to palatal plane angle (OP-PP angle). *Note* O.D.I.=AB-MP angle + PP-FH angle which is not shown in the figure

**Fig. 3:**
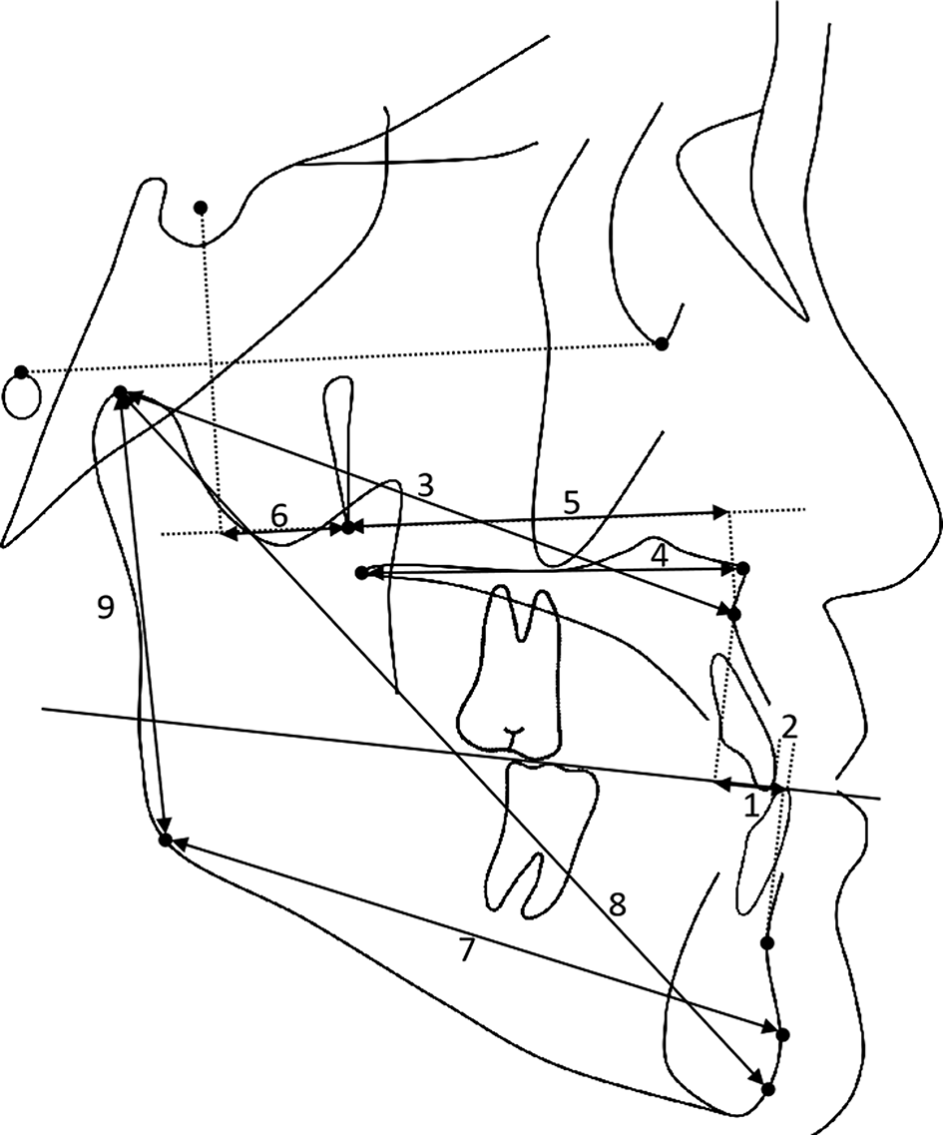
Linear measurements used in this study (1) Intermaxillary position to occlusal plane (Wits appraisal); (2) Overjet; (3) Intermaxillary length (Co-A); (4) Extent of maxillary base (ANS-PNS); (5) Maxillary size (Ptm-A); (6) Maxillary position (Ptm-S); (7) Extent of mandibular base (Pog-Go); (8) Total mandibular length (Gn-Co); (9) Extent of mandibular ramus (Co-Go)

Cephalometric analysis for the three study time points (T0/T1/T2) included the cephalometric measurements of Jarabak, Wits appraisal, Downs, Tweed, Bjork and Johnson, generating 28 measurement items (Figs. 1, 2 and 3).

### Statistical methods

Descriptive statistics at the three study time points (T0/T1/T2) and the corresponding intervals (T0-T1, T1-T2, T0-T2) were calculated. The chi-square test was used to compare the sex proportions in each group (Table 1). The Shapiro-Wilk test was used for normal distribution testing and homogenity of variance were checked by the Levenes test. Data was analyzed using non-parametric tests (Mann-Whitney U test) when they did not show normal distribution and when the variance was not homogeneous. The following comparisons were performed by One Way ANOVA tests: (1) comparison of the mean age ranges and mean durations of the observation intervals for the three groups (Table 2); (2) starting forms of observation (T1) between the treatment group and control groups (Table 4); and (3) comparison of observation duration changes (T1-T2) between the treatment group and control groups (Table 5).

**Table 2:** Mean age range and mean duration of intervals for the treatment group and control groups

	TG	CG III	CG I	ANOVA test P value	
				TG-CG III	TG-CG I
T0 (y)	8.79 ± 1.65				
T0-T1 (y)	1.16 ± 0.52				
T1 (y)	9.95 ± 1.67	9.64 ± 2.53	9.28 ± 0.96	0.933	0.476
T1-T2 (y)	2.05 ± 0.39	2.39 ± 1.29	1.97 ± 0.49	0.867	0.997
T2(y)	12.00 ± 1.75	12.03 ± 3.26	11.25 ± 1.19	1.000	0.378

*P < 0.05; **P < 0.01; ***P < 0.001

**Table 3:** Comparison of active treatment changes and observation duration changes of the treated group

Cephalometric measures	TG Difference (T1-T0)		TG Difference (T2-T1)		Mann‒Whitney U test P value	TG Difference (T2-T0)	
	Mean	SD	Mean	SD		Mean	SD
Sagittal skeletal							
SNA (°)	2.52	2.89	0.15	2.94	0.003^**^	2.68	3.53
SNB (°)	-1.30	2.41	1.79	2.57	0.000^***^	0.49	3.11
ANB (°)	3.82	1.19	-1.62	1.40	0.000^***^	2.20	1.41
Wits (mm)	3.98	2.05	-2.05	2.08	0.000^***^	1.92	2.17
BAr-AAr (°)	-2.14	1.52	2.86	2.42	0.000^***^	0.74	1.85
Vertical skeletal							
ODI (°)	5.47	4.60	-2.18	3.56	0.000^***^	3.29	5.41
Gonial (°)	-0.31	3.52	-0.35	2.32	0.811	-0.65	2.91
MP-SN (°)	1.80	2.01	-1.24	2.36	0.000^***^	0.57	2.70
MP-FH (°)	3.08	2.25	-2.13	1.99	0.000^***^	0.96	2.43
Y-axis (°)	1.63	1.89	-0.69	2.11	0.000^***^	0.95	2.47
Maxillary skeletal							
NA-FH (°)	1.24	2.80	1.05	2.47	0.573	2.30	3.22
Co-A (mm)	3.16	2.09	3.05	2.80	0.627	6.28	3.73
ANS-PNS (mm)	2.88	2.16	1.41	1.95	0.004^**^	4.14	2.17
PP-SN (°)	-0.73	4.23	1.45	2.70	0.005^**^	0.72	4.70
PP-FH (°)	1.08	4.14	0.19	2.78	0.517	1.27	4.34
Ptm-A (mm)	2.29	1.65	0.77	2.50	0.004^**^	3.10	2.17
Ptm-S (mm)	-0.73	1.53	1.42	1.67	0.000^***^	0.71	1.75
Mandibular skeletal							
SNP (°)	-0.98	2.37	1.73	2.61	0.000^***^	0.75	3.03
NP-FH (°)	-2.28	2.44	2.63	2.18	0.000^***^	0.35	2.55
Pog-Go (mm)	1.41	2.23	3.99	2.98	0.001^**^	5.46	3.87
Gn-Co (mm)	1.44	2.55	8.51	5.22	0.000^***^	10.04	6.14
Co-Go (mm)	0.12	2.34	5.65	3.76	0.000^***^	5.83	4.13
Dentoalveolar							
U1-SN (°)	4.69	4.56	0.94	5.85	0.015^*^	5.63	6.52
L1-MP (°)	-1.19	9.00	1.57	3.30	0.000^***^	0.32	8.17
U1-L1 (°)	-3.70	6.88	-1.27	6.44	0.000^***^	-4.91	6.32
OP-MP (°)	1.95	2.99	-0.03	1.84	0.003^**^	1.93	2.73
OP-PP (°)	0.57	5.11	-2.65	3.23	0.000^***^	-2.07	5.88
Overjet (mm)	5.74	2.30	-1.58	1.59	0.038^*^	4.17	2.05

*P < 0.05; **P < 0.01; ***P < 0.001

All statistical computations were performed with SPSS software (version 21.0), and differences were considered statistically significant if P < 0.05. Figures were prepared using GraphPad Prism software (version 8.0.1).

### Power analysis

Post Hoc Power Analyses were performed using G*Power software (version 3.1.9.7) to achieve empirical validity. The input parameters were sample size of the three independent groups, with threshold of significance (alpha,α) of 0.05 which would have a 90% power (1-β) to reject the Null Hypothesis. The effect size was calculated using the partial eta square statistic: 0.01 < η^2^ < 0.05, a small effect size; 0.06 < η^2^ < 0.13, a moderate effect size; and η^2^>0.14, a large effect size. For measurement items where the parametric assumptions were not met, Mann-Whitney Utest was used to calculate the non-parametric effect size which were expressed as Z value.

## Results

There was no significant difference in the sex distribution, mean age range in T1, or mean duration of observation interval between the treatment group and control groups (P > 0.05), as reported in Tables 1 and 2.

**Fig. 4:**
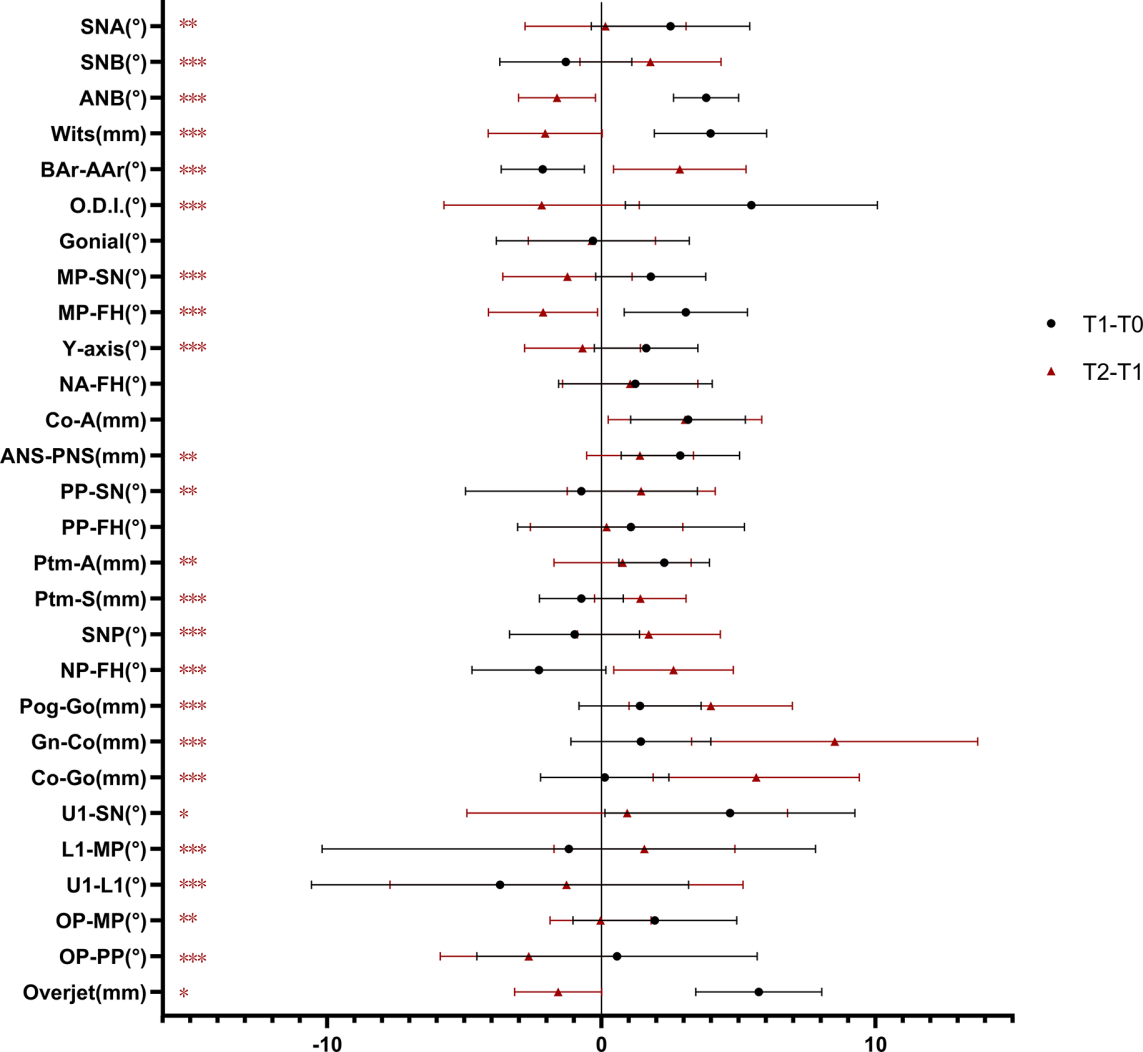
Comparison of active treatment changes and observation duration changes in the treated group *P < 0.05; **P < 0.01; ***P < 0.001.● represents TG difference (T1-T0); ▲ represents TG difference (T2-T1)

Table 3 reports the treatment effects (T0-T1) and growth changes over the observation duration (T1-T2) in the TG, in which the active orthopedic treatment effect showed a opposite trend to the natural craniomaxillofacial growth effect after treatment in many aspects: the T0-T1 changes in TG showed a significantly increase in ANS-PNS and Ptm-A (P < 0.01) and the sagittal maxillomandibular skeletal relation showed highly significant improvements in ANB, Wits appraisal and BAr-AAr (P < 0.001); on the other hand, the T1-T2 changes in TG showed a diminished or even opposite effect to those of T0-T1 (Fig. 4), along with a significantly increase in Pog-Go (P < 0.01), Gn-Co and Co-Go (P < 0.001). Notably, however, NA-FH (P = 0.573) and Co-A (P = 0.627) was not significantly different between the two periods.

**Table 4:** Starting forms of observation (T1) between the treated group and control groups

Cephalometric measures	TG (T1)		CG III (T1)		CG I (T1)		ANOVA test Bonferroni post-hoc test P value		Effect size	
	Mean	SD	Mean	SD	Mean	SD	TG-CG III	TG-CG I	η^2^	
									^†^Z^TG−CG III^	^†^Z^TG−CG I^
Sagittal skeletal										
SNA (°)	80.65	3.11	78.57	4.01	79.24	3.42	0.185	0.467	0.060	
SNB (°)	78.62	3.35	78.89	4.56	75.89	3.42	1.000	0.028^**^	0.121	
ANB (°)	2.03	1.66	-0.36	2.24	3.34	1.16	^†^0.001^**^	^†^0.004^**^	^†^−3.439	^†^−2.865
Wits (mm)	-4.15	2.19	-6.79	3.32	-1.96	1.67	^†^0.008^**^	^†^0.001^**^	^†^−2.653	^†^−3.429
BAr-AAr (°)	14.74	3.35	16.26	4.07	10.11	2.02	0.377	0.000^***^	0.395	
Vertical skeletal										
ODI (°)	69.58	5.06	66.22	6.04	74.25	6.43	0.193	0.014^**^	0.226	
Gonial (°)	128.48	5.86	127.97	3.51	127.05	4.74	1.000	0.929	0.016	
MP-SN (°)	38.13	4.64	36.58	6.29	37.01	4.03	0.926	1.000	0.019	
MP-FH (°)	30.77	4.62	28.74	5.14	27.92	3.85	0.454	0.077	0.078	
Y-axis (°)	71.00	3.37	70.06	4.35	71.54	3.30	1.000	1.000	0.024	
Maxillary skeletal										
NA-FH (°)	88.01	2.67	86.40	3.21	88.33	3.25	0.261	1.000	0.062	
Co-A (mm)	74.53	3.71	73.14	6.71	72.58	3.98	^†^0.387	^†^0.080	^†^−0.865	^†^−1.750
ANS-PNS (mm)	43.24	2.39	45.46	12.41	42.29	2.47	^†^0.854	^†^0.136	^†^−0.185	^†^−1.490
PP-SN (°)	10.60	3.44	10.08	3.78	11.29	4.13	1.000	1.000	0.015	
PP-FH (°)	3.48	3.11	2.22	3.01	2.19	3.33	0.619	0.451	0.040	
Ptm-A (mm)	41.56	2.06	40.89	4.31	40.49	2.41	^†^0.344	^†^0.134	^†^−0.946	^†^−1.499
Ptm-S (mm)	16.88	2.32	16.16	2.43	17.26	1.86	0.878	1.000	0.035	
Mandibular skeletal										
SNP (°)	78.86	3.63	78.84	4.94	76.11	3.60	1.000	0.044^*^	0.100	
NP-FH (°)	86.22	3.22	87.70	3.76	85.16	2.97	^†^0.782	^†^0.290	^†^−0.277	^†^−1.059
Pog-Go (mm)	67.58	3.85	67.53	6.93	63.47	3.53	1.000	0.007^**^	0.152	
Gn-Co (mm)	102.87	5.59	102.33	10.92	96.57	4.20	^†^0.400	^†^0.000^***^	^†^−0.842	^†^−3.725
Co-Go (mm)	50.42	3.53	50.08	6.50	48.47	2.83	^†^0.368	^†^0.043^*^	^†^−0.900	^†^−2.028
Dentoalveolar										
U1-SN (°)	109.98	5.94	103.46	8.44	103.04	7.23	^†^0.017^*^	^†^0.001^**^	^†^−2.387	^†^−3.374
L1-MP (°)	84.43	7.41	87.24	8.99	90.47	5.51	^†^0.362	^†^0.002^**^	^†^−0.911	^†^−3.096
U1-L1 (°)	127.79	10.71	132.71	11.55	129.50	11.14	0.464	1.000	0.030	
OP-MP (°)	17.66	3.38	15.70	4.66	14.89	3.93	0.322	0.037^*^	0.097	
OP-PP (°)	9.52	3.65	10.81	3.78	10.81	3.64	0.792	0.631	0.031	
Overjet (mm)	3.78	1.66	-0.88	2.02	3.98	1.11	^†^0.000^***^	^†^0.829	^†^−5.031	^†^−0.216

*Note* Bonferroni-corrected P values, which were calculated from ANOVA test, are reported in the table; The signs ^†^ represent value performed by Mann‒Whitney U test; *P < 0.05; **P < 0.01; ***P < 0.001

Table 4 shows the T1 values of maxillary development and position and intermaxillary relation in TG intermediate between the two control groups. Meanwhile, there was no significant difference between TG and CG III in Pog-Go, Gn-Co and Co-Go (P > 0.05).

**Table 5:** Analysis of posttreatment effects between the treated group and control groups

Cephalometric measures	TG Difference (T2-T1)		CG III Difference (T2-T1)		CG I Difference (T2-T1)		ANOVA test Bonferroni post-hoc test P value		Effect size	
	Mean	SD	Mean	SD	Mean	SD	TG-CG III	TG-CG I	η^2^	
									^†^Z^TG−CG III^	^†^Z^TG−CG I^
Sagittal skeletal										
SNA(°)	0.15	2.94	-0.09	2.00	1.45	1.26	^†^0.346	^†^0.108	^†^−0.943	^†^−1.607
SNB (°)	1.79	2.57	2.28	2.55	0.39	1.01	^†^1.000	^†^0.000^***^	^†^−0.000	^†^−3.615
ANB (°)	-1.62	1.40	-2.32	1.46	1.08	0.84	0.136	0.000^***^	0.574	
Wits (mm)	-2.05	2.08	-1.37	3.84	1.87	1.56	1.000	0.000^***^	0.339	
BAr-AAr (°)	2.86	2.42	4.02	2.53	-0.79	1.11	^†^0.119	^†^0.000^***^	^†^−1.558	^†^−5.121
Vertical skeletal										
O.D.I. (°)	-2.18	3.56	-3.19	3.66	1.06	2.79	1.000	0.001^**^	0.238	
Gonial (°)	-0.35	2.32	2.42	1.83	0.30	2.45	0.001^**^	1.000	0.184	
MP-SN (°)	-1.24	2.36	1.83	2.18	-0.46	1.45	0.000^***^	0.466	0.271	
MP-FH (°)	-2.13	1.99	1.63	1.62	-0.59	1.36	0.000^***^	0.003^**^	0.459	
Y-axis (°)	-0.69	2.11	1.58	1.99	-0.01	1.04	0.000^***^	0.432	0.213	
Maxillary skeletal										
NA-FH (°)	1.05	2.47	-1.55	1.40	1.60	1.68	0.000^***^	1.000	0.325	
Co-A (mm)	3.05	2.80	2.69	1.19	4.60	2.04	^†^0.414	^†^0.004^**^	^†^−0.817	^†^−2.892
ANS-PNS (mm)	1.41	1.95	1.15	1.59	2.46	1.53	^†^0.870	^†^0.006^**^	^†^−0.164	^†^−2.737
PP-SN (°)	1.45	2.70	0.19	3.96	-0.47	2.73	0.873	0.170	0.058	
PP-FH (°)	0.19	2.78	1.33	3.71	-0.47	2.05	^†^0.152	^†^0.815	^†^−1.432	^†^−0.234
Ptm-A (mm)	0.77	2.50	0.54	1.25	2.58	1.29	^†^0.792	^†^0.001^**^	^†^−0.264	^†^−3.224
Ptm-S (mm)	1.42	1.67	0.73	2.41	0.22	1.09	^†^0.113	^†^0.008^**^	^†^−1.584	^†^0.077
Mandibular skeletal										
SNP (°)	1.73	2.61	2.64	2.41	0.54	1.04	^†^0.792	^†^0.002^**^	^†^−0.264	^†^−3.116
NP-FH (°)	2.63	2.18	1.48	1.73	0.73	1.25	0.035^*^	0.001^**^	0.242	
Pog-Go (mm)	3.99	2.98	5.30	2.23	2.43	1.74	^†^0.013^*^	^†^0.149	^†^−2.488	^†^−1.442
Gn-Co (mm)	8.51	5.22	10.01	3.91	4.14	2.36	0.763	0.003^**^	0.249	
Co-Go (mm)	5.65	3.76	6.26	2.35	2.17	1.79	1.000	0.000^***^	0.281	
Dentoalveolar										
U1-SN (°)	0.94	5.85	8.07	4.72	1.65	9.04	0.002^***^	0.659	0.168	
L1-MP (°)	1.57	3.30	-2.01	4.72	1.20	5.16	^†^0.006^**^	^†^0.915	^†^−2.727	^†^−0.107
U1-L1 (°)	-1.27	6.44	-5.78	6.32	-2.41	11.98	^†^0.063	^†^0.733	^†^−2.224	^†^−0.224
OP-MP (°)	-0.03	1.84	2.41	2.52	1.35	2.94	^†^0.002^**^	^†^0.092	^†^−3.143	^†^−1.685
OP-PP (°)	-2.65	3.23	-3.44	4.23	-1.32	3.62	1.000	0.753	0.051	
Overjet (mm)	-1.58	1.59	-0.12	2.09	0.37	1.83	0.022^*^	0.000^***^	0.218	

*Note* Bonferroni-corrected P values, which were calculated from ANOVA test, are reported in the table; The signs ^†^ represent value performed by Mann‒Whitney U test; *P < 0.05; **P < 0.01; ***P < 0.001

Table 5 reports the posttreatment effects by comparing the T1-T2 changes in TG with the two control groups. Decrease in ANB, Wits appraisal and BAr-AAr in TG and CG III were statistically significant compared to CG I (p < 0.001). The mean value of ANS-PNS and Ptm-A in TG was larger than that in CG III, but the difference was not statistically significant (P > 0.05). Meanwhile, TG and CG I showed a significant increase in NA-FH (P < 0.001) compared to CG III. TG presented a significant increase in Gn-Co (P < 0.01) and Co-Go (P < 0.001), except for changes in the extent of the mandibular base (Pog-Go, P = 0.149) compared to CG I. The vertical maxillomandibular skeletal variables (Gonial; MP-SN; MP-FH; Y-axis) in treatment group decreased significantly compared to those in CG III (P < 0.01). U1-SN and L1-MP showed a significant increase, which was similar to CG I (P > 0.05), and Overjet decreased significantly relative to both of the two control groups (P < 0.05). OP-MP mildly decreased in TG, which significantly differed from that in CG III (P < 0.01).

The stable rate in TG was 77.42% who demonstrated a positive overjet and a class I or close to class III molar relationship at T2.

## Discussion

Due to the unpredictable growth patterns and the propensity for relapse of Class III subjects, growth after maxillary protraction has received close attention in several studies [15–17]. A tendency of reestablishment of the skeletal Class III growth pattern was observed in our study, as presented by the T1-T2 changes in ANB (-1.62°), Wits appraisal (-2.05 mm) and BAr-Aar (2.86°) in TG, which was generally similar to untreated Class III subjects. Previous studies have indicated that the sagittal maxillomandibular skeletal relation after active protraction therapy is mainly affected by the growth rate and sagittal position changes of the mandible [4, 15, 18]. Similarly, this study found that there was a rise of the growth rate in the extent of the mandibular base and ramus and total mandibular length (Pog-Go, 3.99 mm; Co-Go, 5.65 mm; Gn-Co, 8.51 mm) between T0-T1 and T1-T2. Parts of the rise could be explained by the natural growth potential of the mandible, as has been described by the values in CG I. The recovery growth may explain another part of this recurrence when maxillary protraction use was discontinued before facial growth was complete [15, 17]. Untreated Class III subjects tend to have a longer mandibular growth spurt than class I adolescents, which lasts until young adulthood, especially in males [5, 19]. This raised the possibility of relapse if there is a rebound of the mandible growth after the end of maxillary protraction. Thus, the restriction to mandibular growth is the critical factor for obtaining favorable intermaxillary outcomes. When lifting the restriction from the counterforce of the protraction facemask to the menton, however, the changes in the extent of the mandibular base, which were as large as those in subjects with normal occlusion (CG I) [20], did not show this trend of growth faster. Indicating that maxillary protraction did have a certain degree of postimpact on mandibular growth. This might be due to changes in the direction of condylar growth, as reported in the literature: untreated Class III subjects tend to show an upward and backward direction of condylar growth, and the treated group tends to present an upward and forward direction of condylar growth [21, 22]. This uneven growth trend of various parts of the mandible thus promoted differences in the vertical facial pattern and inclination of the occlusal plane [23].

The reduction in the growth rate of the maxilla partly contributed to the skeletal discrepancy, since the excess growth trend of the mandible cannot be matched by the maxilla [24]. As argued by Lee et al., no significant difference was observed in the SNA in the treated groups more than 3 years after protraction therapy when compared with untreated Class III subjects [25]. With respect to the former, the difference between T0-T1 and T1-T2 in terms of maxillary growth (the changes in ANS-PNS and Ptm-A) in TG suggested that the accelerated maxillary growth in T0-T1 is a kind of adaptive change under orthopedic forces, and the pattern of maxillary growth in Class III subjects when ceasing the treatment may not be fundamentally changed by protraction therapy. Remarkably, the advancement of the maxilla may be one long term effect [26]. We found that NA-FH both at T0-T1 (1.24°) and T1-T2 (1.05°) in TG exhibited significant increases similar to those in CG I, recapitulating the findings from previous studies [12, 16, 27]. The great potential of the maxilla for advancement after treatment may be partly explained by the opening of the transversal palatal suture under the anterior directed force during treatment, which acts by the conduction of corrected maxillomandibular stress in the approximate Class I direction [28, 29].

Growth rotation can be expected in the process of posttreatment growth since the structural relations of the craniomaxillofacial region tend to restabilize after treatment and lead to a balance between treatment outcomes and growth [30]. We found that the maxilla in TG tended to rotate counterclockwise from T0-T1 (SN-PP, -0.98°) and clockwise from T1-T2 (SN-PP, 1.73°) relative to the anterior skull base. Growth rotation of the nasomaxillary complex could assist clinicians in determining whether the therapeutic outcomes have reached the functional stability. Although these results differed from what Kwak et al. found [31], the mean changes were not clinically significant, as they indicated. On the other hand, the clockwise rotation of the mandible in untreated or actively treated (T0-T1) Class III subjects is often addressed in the literature [5, 22], and the rotation in the downward and backward direction could discreetly play a role in masking the overgrowth of the mandible to some extent. However, it is crucial to focus clinical attention on vertical growth in cases of hyperdivergent skeletal class III malocclusions. This is because if there is limited sagittal growth or anterior advancement of the maxilla during treatment, and the sagittal intermaxillary relationship is mainly masked by the clockwise rotational of the mandible, the resulting pseudo Class I relationship is more prone to relapse. Additionally, the mandible in TG tended to rotate counterclockwise (MP-SN, -1.24°; MP-FH, -2.13°; Y-axis, -0.69°), and the gonial angle decreased (-0.35°) from T1-T2, which, in contrast to CG III, presented a compensatory effect for the clockwise rotation at T0-T1. This trend of the growth modification was similar to that found in the literature [22, 32, 33], and the closure of the gonial angle was seen as a favorable mechanism that could limit linear increases of the mandible along Co-Gn [22]. However, whether this direction of mandible rotation is correlated with unfavorable instability of treatment remains controversial [30, 31].

More dentoalveolar compensation was observed in labial tipping in the upper incisors and lingual tipping in the lower incisors in CG III, and a dental physiological mechanism attempting to maintain a positive overjet in TG manifested as slight labial tipping in the upper incisors (U1-SN, 0.94°), but labial tipping in the lower incisors (L1-MP, 1.57°), which was caused by the removal of restricting forces, represented the underlying tendency for skeletal relapse [14]. Thus, the significant decrease in overjet (-1.58 mm) could be attributed to the struggle with skeletal relapse [4]. The most common criteria for class III relapse are class III molar relationship and anterior crossbite. Therefore, a positive overjet was often seen as an evaluation criterion of clinical stability, and 77.42% positive overjet was observed in our study, which is close to that reported in previous studies: Palma et al. observed 81.8% [11], Masucci et al. 73% [18], and Tejedor et al. 73.3% in males and 80% in females [14], but the observation duration and retention protocol reported in these literatures vary.

Overall, although a degree of reestablishment of the skeletal Class III growth pattern over the observation duration can be expected, it is worth noting that the sagittal maxillomandibular skeletal relation outcomes at T1-T2 were counteracted by the active treatment effect (T0-T1), and an increase in ANB (2.20°) and Wits appraisal (1.92 mm) occurred over the total duration (T0-T2). Additionally, previous studies have indicated that early interception of skeletal class III malocclusion may reduce the risk of orthognathic surgery [6, 34], and it is important to determine that what extent outcomes should be achieved in active treatment to better reduce the need for future surgeries [35]. Therefore, it is necessary to inform the patients and their parents that prepuberal class III treatment is a very long treatment that requires overcorrection and a long retention period. However, the question remains whether the advancement of the maxilla and restraining effect on the growth of the mandibular base after maxillary protraction is able to counteract the reestablishment of subsequent growth. Additionally, this study was biased toward immediate posttreatment modifications, and longer longitudinal studies are necessary for observation, especially those containing the pubertal growth spurt. Furthermore, it will be of interest to investigate protocols that could effectively limit mandibular growth that have been described previously, such as chincap therapy and miniplates and the Class III elastics protocol [36].

## Conclusion

1. A tendency of reestablishment of the skeletal Class III growth pattern was observed after maxillary protraction therapy, which was caused by greater protrusion of the mandible relative to the maxilla.
2. Maxillary protraction did have a certain degree of postimpact on mandibular growth, as evidenced by the changes in the extent of the mandibular base, which did not show the trend of growth faster.
3. Protraction therapy does not fundamentally change the mode of maxillary growth in Class III subjects when ceasing the treatment, but advancement in the sagittal direction of the maxilla may be one effect that can last into the long term.
4. The maxilla tended to rotate clockwise relative to the anterior skull base, and the mandible tended to rotate counterclockwise during the observation period.
5. Dentoalveolar compensation was indicated by slight labial tipping in the upper and lower incisors and a decrease in overjet.

## Data Availability

Data and materials are available from the corresponding author on reasonable request.

## List of abbreviations

TG: Treatment group of skeletal Class III malocclusion
CG III: Control group of skeletal class III malocclusion
CG I: Control group of skeletal class I malocclusion
CVS: Cervical stage
RME: Rapid maxillary expansion
